# In Vitro Infection of Human Macrophages with *Porphyromonas gingivalis* W83

**DOI:** 10.3390/ijms26031054

**Published:** 2025-01-26

**Authors:** Martina La Rosa, Alessandra Spagnolo, Juan Daniel Gamonal, Maria Jose Marín, Elena Figuero, Mariano Sanz

**Affiliations:** ETEP (Etiology and Therapy of Periodontal and Peri-Implant Diseases) Research Group, Faculty of Dentistry, Complutense University, 28040 Madrid, Spain; mlarosa@ucm.es (M.L.R.); aspagnol@ucm.es (A.S.); juandang@ucm.es (J.D.G.); mjmarin@ucm.es (M.J.M.); efigueruiz@odon.ucm.es (E.F.)

**Keywords:** *Porphyromonas gingivalis* W83, macrophages, M1 phenotype

## Abstract

This study aimed to investigate the innate immune response of human macrophages to *Porphyromonas gingivalis* W83 using a novel in vitro infection model. The growth kinetics of *P. gingivalis* W83 were analyzed, revealing an exponential growth phase at 8 h (optical density = 0.70). To establish a reliable macrophage model, the differentiation of THP-1 monocytes into macrophages was optimized using low concentrations of phorbol 12-myristate 13-acetate (PMA). This approach induced enhanced adherence and morphological changes, with full differentiation achieved after 48 h of PMA treatment followed by 24 h of rest. Polarization towards the pro-inflammatory M1 phenotype was successfully induced with interferon-γ (IFN-γ) and lipopolysaccharide (LPS), as confirmed using cytokine profiling. Cytokine analysis using Luminex^®^ technology demonstrated significant increases in interleukin (IL)-1β, tumor necrosis factor-α (TNF-α), and IL-6, indicating the effective activation of macrophages towards a pro-inflammatory phenotype. Building upon this macrophage model, this study investigated the interactions between macrophages and *P. gingivalis* W83 during its exponential growth phase. After a one-hour infection period, bacterial DNA quantification in supernatants and lysed macrophages revealed minimal levels of internalized or adherent bacteria, supporting the hypothesis that *P. gingivalis* effectively evades immune detection. These findings emphasize the utility of this model in uncovering the sophisticated immune evasion strategies employed by *P. gingivalis*, with significant implications for the development of targeted therapeutic interventions.

## 1. Introduction

Periodontitis is considered a complex chronic inflammatory disease resulting from the host’s immune response to an unbalanced subgingival biofilm, a phenomenon known as dysbiosis [1]. In this process, the diverse bacterial communities within the biofilm shift from a symbiotic to a dysbiotic state, which fosters the host response towards a catabolic state, which leads to tissue destruction. This microbial imbalance, together with the host’s inability to effectively resolve the inflammatory process, will promote tissue damage and exacerbate the progression of periodontitis [2].

*Porphyromonas gingivalis* W83 is a highly virulent strain extensively studied for its critical role in periodontitis and its association with systemic diseases [3]. As a keystone pathogen, *P. gingivalis* disrupts microbial homeostasis, driving a shift from a symbiotic to a dysbiotic state [4,5]. This transition underlies its ability to orchestrate inflammatory responses that contribute to tissue destruction and systemic dissemination [6,7,8]. W83 harbors several virulence factors, including gingipains (arginine- and lysine-specific proteases), sialidases, lipopolysaccharides (LPS) [9,10,11,12,13], and the novel virulence gene PG0717, which modulates the host’s autophagic pathway, enabling immune evasion and persistence within host cells [14,15]. Additionally, its encapsulation as a K1 serotype enhances its virulence compared to non-encapsulated strains [16,17,18]. The genome sequencing of W83 revealed insights into its metabolism, transport mechanisms, and unique virulence profiles [19,20]. Compared to less virulent strains, such as 33277, W83 exhibits specific gingipain genotypes that are highly correlated with its pathogenicity [6,21]. It also manipulates host immune responses through epigenetic alterations, enhancing survival and pathogenicity [22]. Furthermore, components of its membrane significantly alter the gene expression in host cells, promoting chronic inflammation [23].

The likely interaction of this pathogen with the macrophages, central components of the innate immune system, is of particular significance, since macrophages are among the first immune cells to respond to infection and, hence, they play a critical role in maintaining periodontal tissue homeostasis. Through pattern recognition receptors (PRRs), macrophages detect pathogen-associated molecular patterns (PAMPs) and eliminate pathogens via phagocytosis [24,25,26,27,28]. Classically activated M1 macrophages, induced by signals such as interferon-γ (IFN-γ) and tumor necrosis factor-α (TNF-α), are key players in this response, exhibiting a Th1-type phenotype that promotes pathogen destruction [29,30,31,32]. However, *P. gingivalis* W83 has evolved sophisticated mechanisms to evade macrophage-mediated killing. For instance, it alters macrophage phagocytic capacity by sequestering lipids from the macrophage membrane and uses fimbriae and accessory proteins to disrupt intracellular signaling pathways, suppressing the production of reactive oxygen and nitrogen species critical for pathogen elimination [33,34]. These immune evasion strategies enable *P. gingivalis* W83 to persist in the tissues and, thus, promote chronic inflammation and tissue destruction.

Post-genomic studies have shed light on the essential genes of *Porphyromonas gingivalis* and the M1 macrophage phenotype [35,36,37]. However, the lack of a dedicated in vitro model to study the interactions between *P. gingivalis* W83 and differentiated macrophages has limited the detailed investigation of these processes. Despite significant advances in understanding the pathogenesis of this highly virulent strain, critical gaps persist in unraveling the molecular mechanisms underlying its interactions with and evasion of the macrophage immune response. This study seeks to address these gaps by establishing a tailored platform to investigate the molecular mechanisms employed by strain W83. Through a focus on cytokine profiling, immune evasion strategies, and genomic changes, this model aims to provide a robust framework for advanced research on host–pathogen dynamics.

The null hypothesis (H_0_) posited that *P. gingivalis* LPS does not significantly induce an inflammatory M1 phenotype in THP-1-derived macrophages or alter the levels of pro-inflammatory cytokines (IL-1β, TNF-α, and IL-6).

## 2. Results

### 2.1. Growth Curve Optimization

**Figure 1:**
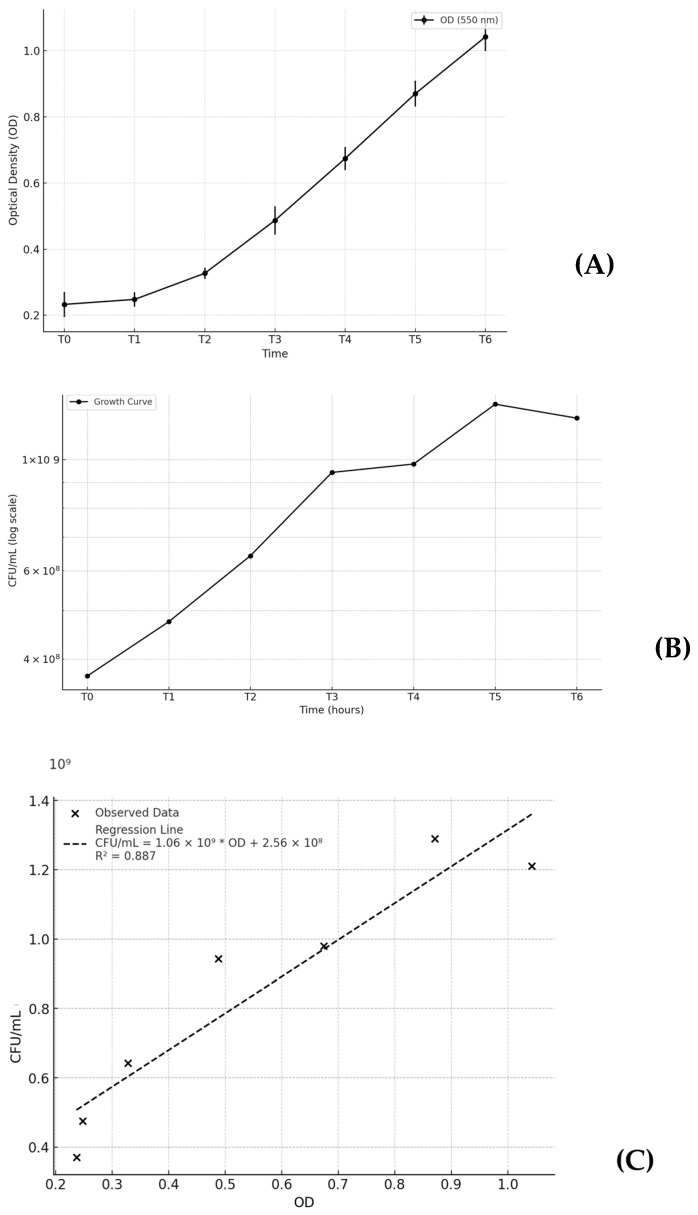
(**A**) Plot showing the evolution of optical density (OD) at 550 nm over time, representing bacterial population growth with standard deviation. (**B**) Plot depicting the change in colony-forming units (CFU/mL) over the experimental time points (T0 to T6), showing the bacterial growth trend. (**C**) Scatter plot with a regression line illustrating the correlation between optical density (OD) and CFU/mL, accompanied by the regression equation.

**Figure 2:**
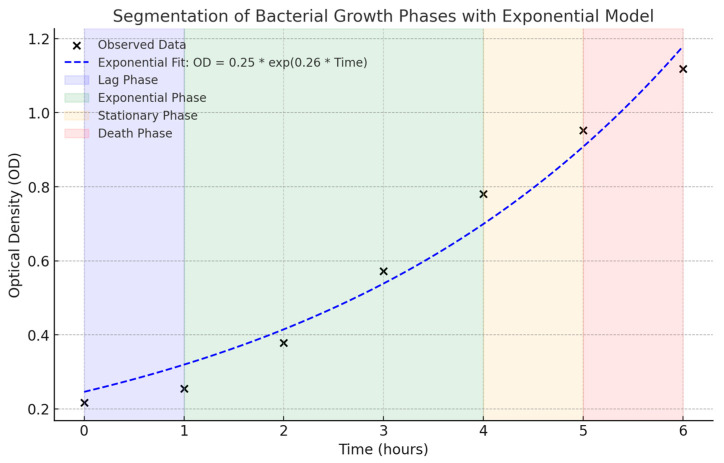
Exponential fit of the growth curve segregating distinct bacterial growth phases (lag phase (blue), exponential phase (green), stationary phase (yellow), and death phase (red)) with data points. x: observed data. * represented × (multiplication sign).

The growth curve is reported in Figure 1. Panel (A) represents the OD as a function of time. Panel (B) shows the trend of CFU/mL over time, and panel (C) illustrates the logistic regression model used to fit the data. The data presented in Table 1 represent the mean values obtained from the same experiment performed with three independent replicates on different days (*n* = 3). Each replicate was conducted under the same experimental conditions. It illustrates the bacterial phases over time, segmented into four characteristic stages: lag, exponential, stationary, and death phases. During the lag phase (T0–T1), the OD increases slightly from 0.23 to 0.24 (Figure 1A), while the CFU/mL rises modestly (3.70 × 10^8^–4.75 × 10^8^) (Figure 1B). The exponential phase (T1–T4) is marked by rapid growth, with ODs increasing from 0.25 to 0.67 (Figure 1A) and the CFU/mL rising steeply from 4.75 × 10^8^ CFU/mL to 9.80 × 10^8^ CFU/mL (Figure 1B). During this phase, the exponential growth model (OD(t) = OD(t_0_) e^k*t^) reflects a straight line on the logarithmic scale (Figure 2), with a growth rate constant (K) of 0.26. The linear regression analysis (Figure 1C) between the OD and the CFU/mL revealed a strong and statistically significant relationship. The slope of 1.06 × 10^9^ indicates that for each unit increase in the OD, the CFU/mL increases by approximately 1.06 × 10^9^ with an intercept of 2.56 × 10^8^. The correlation coefficient (R = 0.942) and R^2^ = 0.887 show that the model explains 88.7% of the variability in the data, highlighting the OD as a reliable predictor of the CFU/mL. The *p*-value of 0.0015 confirms the relationship is statistically significant (*p* < 0.05).

### 2.2. Quantitative PCR (qPCR) Standard Curve:

The relationship between the Cq value and the logarithm of DNA concentration was described using the equation Cq = 41.309 − 3.3637 × log (DNA). The coefficient of determination (R^2^ = 0.9988) indicated a highly consistent linear relationship, with 99.88% of the variation in Cq values accounted for by changes in the DNA concentration. The efficiency of the qPCR reaction was calculated using the slope of the standard curve with the formula 10^(−1/slope)^, yielding an efficiency of approximately 98%, which falls within the optimal range of 90–100%.

### 2.3. Macrophage Activation and Polarization

#### 2.3.1. Differentiation of THP-1 Cells Into Macrophage Using PMA

Initially, THP-1 cells were seeded in a 48-well plate at a concentration of 1 × 10^5^ cells/mL or 2 × 10^5^ cells/mL. PMA was added to these cells at concentrations of 25, 50, or 100 ng/mL. PMA was removed after 24 h, and phase-contrast images were taken 24 h later (rest time). Additionally, untreated controls without PMA were included in the plate.

**Figure 3:**
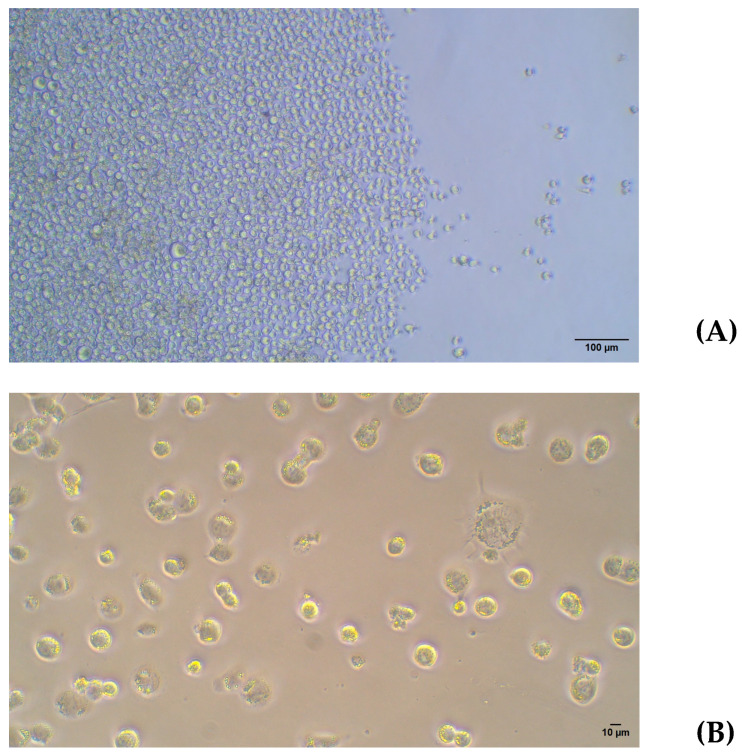
(**A**) THP-1. 10×. (**B**) THP-1 with PMA 25 ng/mL for 24 h and 24 h rest time. Light microscopy images 20×. PMA: phorbol 12-myristate 13-acetate.

The lowest concentration of PMA (25 ng/mL) was enough to induce cell adhesion to the plate and for developing the typical flattened macrophage morphology with the appearance of laminar evaginations, as shown in Figure 3.

**Figure 4:**
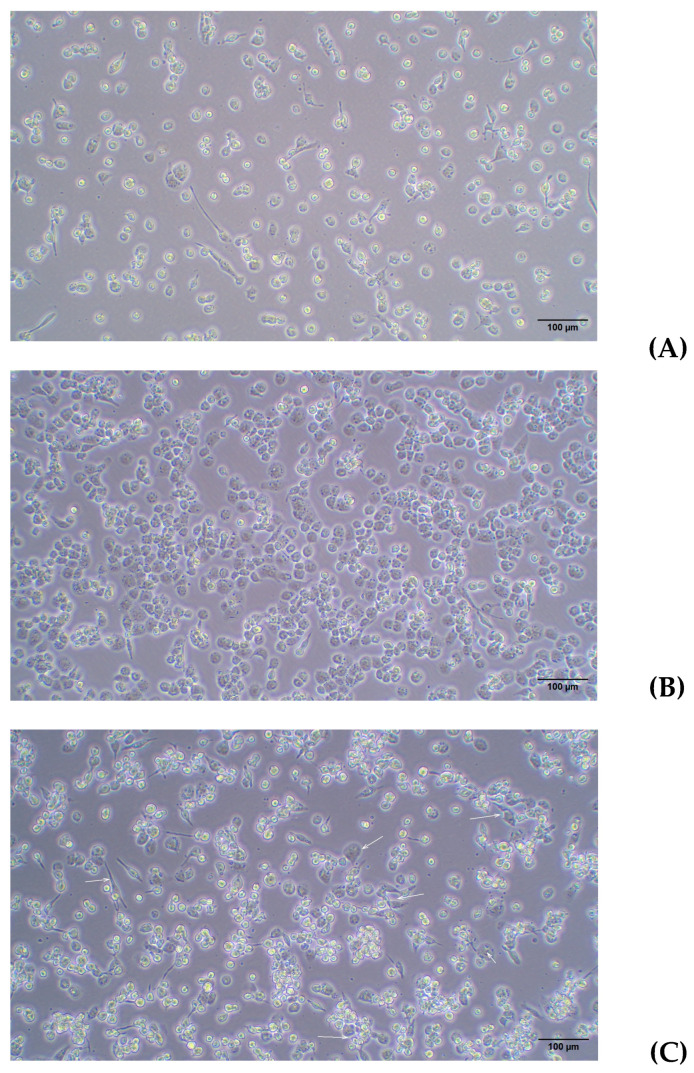
(**A**) A total of 2 × 10^5^ cells/w with PMA for 24 h; (**B**) 2 × 10^5^ cells/w with PMA for 48 h; (**C**) 2 × 10^5^ cells/w with PMA for 48 h and 24 h of rest. Light microscopy images 10×. PMA: phorbol 12-myristate 13-acetate.

**Figure 5:**
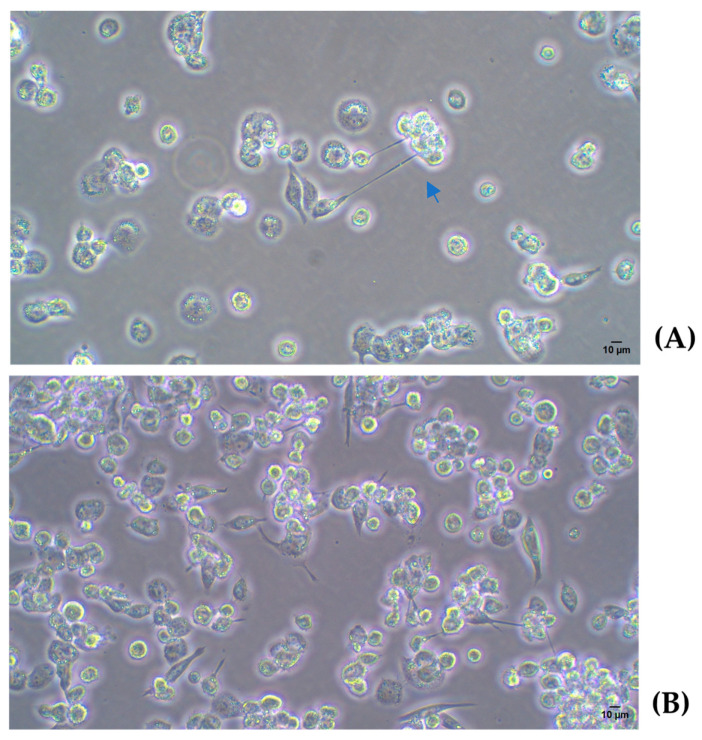
(**A**) A total of 2 × 10^5^ cells/w with PMA for 24 h; (**B**) 2 × 10^5^ cells/w with PMA for 48 h. Light microscopy images 20×. PMA: phorbol 12-myristate 13-acetate.

Next, the lowest concentration of PMA (25 ng/mL) was evaluated at different time points to identify the optimal conditions for THP-1 differentiation into macrophages. As shown in Figure 4, the PMA treatment for 48 h allowed a higher number of cells to attach to the plate, with a clear trend to lose the round morphology and a greater intercellular adhesion when compared to the 24 h treatment. Finally, if PMA was removed and cells were kept for 24 h without further stimulus (rest condition), fewer adhered cells appeared, while a higher number of differentiated macrophages with spindle, amoeboid shapes, and cytoplasmic connections were observed. The high magnification images provide greater detail of the morphology of THP-1 cells differentiated into macrophages following PMA treatment. Figure 5 shows a progressive increase in cell differentiation after 24 h (A) and 48 h of treatment (B). Greater cell adhesion is observed, along with a clear trend toward forming intercellular connections (Blue arrow Figure 5A), which further supports the role of extended treatment.

To eliminate the “well effect” responsible for the different distribution of cells in the center compared to the edges of the well in a microplate, T25 flasks (25 cm^2^ adherent growth surface) were selected. Therefore, THP-1 cells were seeded at the previously tested cell density (2 × 10^5^ cells/mL) and at higher concentration (4 × 10^5^ cells/mL). All cells were incubated with 25 ng/mL of PMA for up to 72 h.

**Figure 6:**
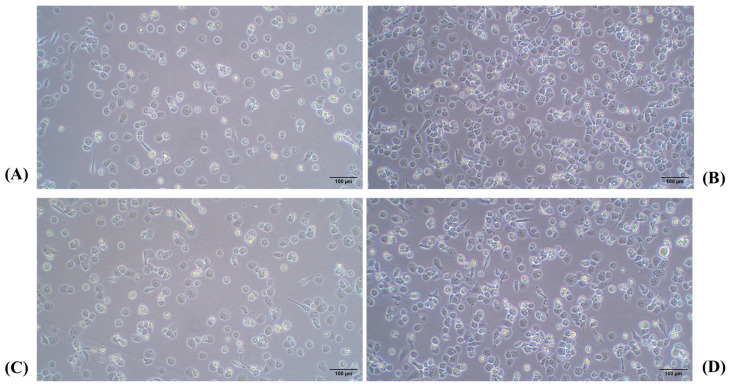
(**A**) A total of 2 × 10^5^ cells/mL with PMA for 48 h; (**B**) 4 × 10^5^ cells/mL with PMA for 48 h; (**C**) 2 × 10^5^ cells/mL with PMA for 72 h; (**D**) 4 × 10^5^ cells/mL with PMA for 72 h. Light microscopy images 10×. PMA: phorbol 12-myristate 13-acetate.

**Figure 7:**
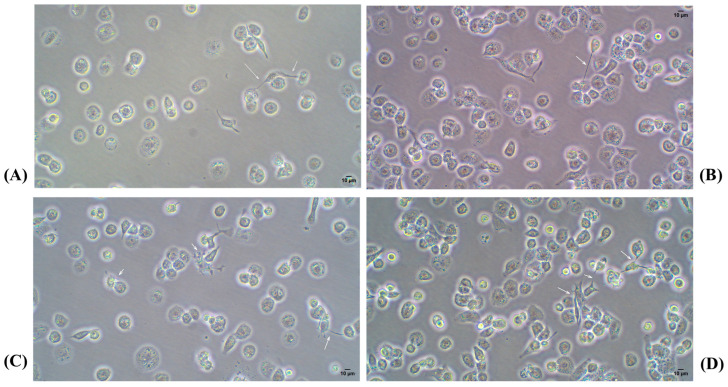
(**A**) A total of 2 × 10^5^ cells/mL with PMA for 48 h; (**B**) 4 × 10^5^ cells/mL with PMA for 48 h; (**C**) 2 × 10^5^ cells/mL with PMA for 72 h; (**D**) 4 × 10^5^ cells/mL with PMA for 72 h. Light microscopy images 20×. PMA: phorbol 12-myristate 13-acetate.

**Figure 8:**
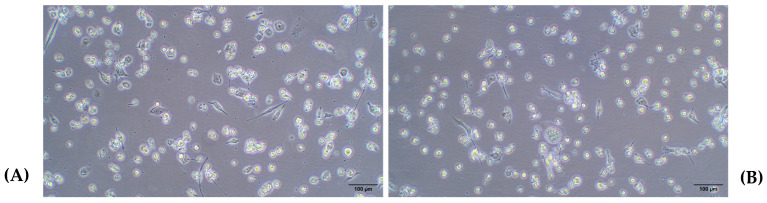
(**A**) A total of 2 × 10^5^ cells/w with PMA for 48 h and conditioned medium; (**B**) 2 × 10^5^ cells/w with PMA for 48 h with wash and change medium. Light microscopy images 10×. PMA: phorbol 12-myristate 13-acetate.

As shown in Figure 6, no evident changes were observed between 48 and 72 h at both cell densities (a–c and b–d). Additionally, the high-magnification images (Figure 7) highlight the maintenance of the characteristic flattened and elongated morphology, as well as the development of cytoplasmic connections, confirming the differentiation process under the given conditions. Moreover, it was confirmed that after a 24-h resting period, macrophages lose adhesion, especially if washing is performed before changing the medium, as seen in Figure 8.

**Figure 9:**
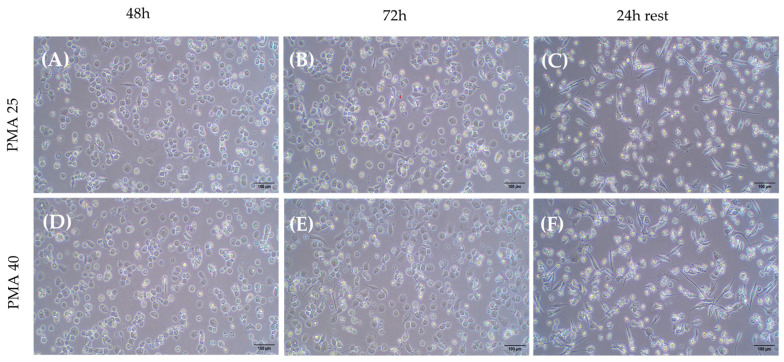
(**A**) A total of 25 ng/mL PMA for 48 h; (**B**) 25 ng/mL PMA for 72 h; (**C**) 25 ng/mL PMA with 24 h of rest; (**D**) 40 ng/mL PMA for 48 h; (**E**) 40 ng/mL PMA for 72 h; (**F**) 40 ng/mL PMA with 24 h of rest. Light microscopy images 10×. PMA: phorbol 12-myristate 13-acetate.

For a higher cell density (4 × 10^5^ cells/mL), a higher concentration of PMA (40 ng/mL) was maintained for up to 72 h, followed by 24 h rest. As shown in Figure 9, there were no evident changes from 48 to 72 h (Figure 7A–D) or between 25 and 40 ng/mL PMA (Figure 7A–C and Figure 7B–D). A loss of adhesion in some cells was confirmed, and a greater number of elongated macrophages were observed in Figure 9C–F.

#### 2.3.2. M1 Polarization

The data from the Luminex^®^ assay are presented as the fold-change of the pro-inflammatory cytokines IL-1β, TNF-α, and IL-6 production under different experimental conditions, normalized to the baseline group (No Stimulated PMA48 h_24 hrest). The conditions included treatment with IFN-γ (20 ng/mL) and various concentrations of LPS (1, 10, 100 ng/mL), both alone and in combination with IFN-γ. Values marked with * correspond to a second independent experiment conducted using fresh (non-frozen) samples.

**Figure 10:**
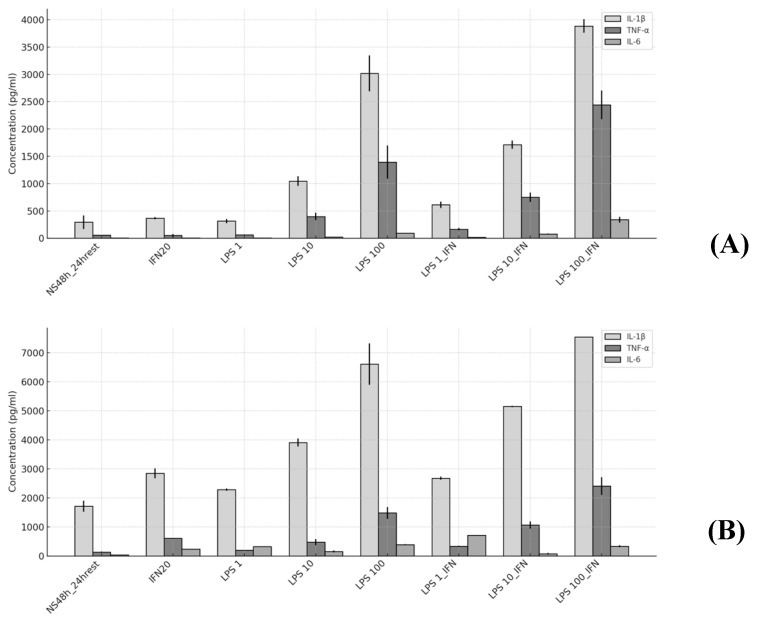
Levels of pro-inflammatory cytokines IL-1β, TNF-α, and IL-6 (pg/mL) in two independent experiments: Experiment 1 (**A**) and Experiment 2 (**B**). Analyte concentrations (pg/mL) are presented as means of two technical replicates, with standard deviations.

In the first experiment (Figure 10A), IL-1β levels exceeded 3500 pg/mL under the LPS (100 ng/mL) + IFN-γ condition, showing a progressive increase with LPS concentration, with a relative increase of up to 13.26 times the control under the same condition. In the second experiment (Figure 10B), a maximum value of nearly 8000 pg/mL is observed, also indicating a strong response (~4.38-times higher than the control).

For TNF-α, a dose-dependent behavior was observed in both experiments, with a maximum of approximately 3000 pg/mL under the LPS (100 ng/mL) + IFN-γ condition in the first experiment. The relative increase for this condition was 41.83 times the control (Figure 10A).

For IL-6, the first experiment (Figure 10A) shows more modest values (~400 pg/mL), increasing under the combination of LPS and IFN-γ, corresponding to 49.93 times the control. In the second experiment (Figure 10B), IL-6 levels reach approximately 700 pg/mL, also reflecting an increase under the same conditions (8.26 times the control).

Across all cytokines (IL-1β, TNF-α, and IL-6), the experimental groups stimulated with LPS 100 ng/mL and LPS 100 ng/mL + IFN-γ exhibited the most pronounced effects. Among these, TNF-α consistently shows the largest fold increases, followed by IL-6 and IL-1β. IL-4 was not detected in the Luminex^®^ assay, consistent with its characteristic association with the M2 macrophage phenotype.

### 2.4. Infection Assay

**Figure 11:**
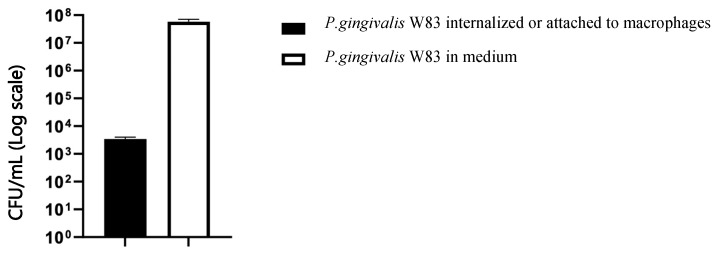
Logarithmic bar chart with distribution of *P. gingivalis* bacteria after 1 h of incubation. Data are represented as means with standard deviations from three independent experimental replicates (*n* = 3).

After a one-hour incubation of unprimed macrophages with *P. gingivalis*, bacterial cells were centrifuged from media and lysed macrophages, and microbial DNA was purified. As shown in Figure 11, the quantification of specific *P. gingivalis* amplicons using q-PCR from DNA samples obtained after lysis of infected macrophages revealed low amounts of *P. gingivalis* bacteria cells, either internalized or adhered, while no amplification was detected in mock-infected macrophages. On the other hand, it was confirmed that the levels of *P. gingivalis* bacteria cells from the supernatants of infected macrophages agreed with the initial inoculum.

## 3. Discussion

A preliminary in vitro model of human macrophage infection was developed using the THP-1 cell line for its methodological advantages (fast expansion rate, possibility of storing in liquid nitrogen, homogeneous genetic background, and well-established differentiation protocols) and the *P. gingivalis* W83 strain for its high virulence factors (gingipains, LPS, PG0717 gene, K1 serotype, and capsule) focusing on short-term interactions (1 h) under aerobic conditions. This approach provides new insights into the pathogenesis of periodontitis and helps identify potential therapeutic targets. Our findings reject the null hypothesis, demonstrating a marked pro-inflammatory response characterized by significant increases in cytokines IL-1β, TNF-α, and IL-6 when macrophages were exposed to *P. gingivalis*-LPS. The observed trends in the cytokine levels indicate that LPS effectively polarizes macrophages toward an M1 phenotype, even though the data from the two experiments could not be combined due to differences in the storage methods of the analyzed cytokines (fresh vs. stored at −80 °C). This consistency becomes evident when the analyte concentrations are normalized to the baseline group (NS 48 h_24 h rest), as shown in Table 2.

The observed *P. gingivalis* growth dynamics align well with the expected behavior of these bacterial populations. The growth rates observed are consistent with prior findings for similar conditions, although the slight drop in CFU/mL during the stationary phase could differ depending on the strain or medium used. For example, some studies reported prolonged stability during the stationary phase [3], while others noted an earlier cell death due to the release of metabolic byproducts [38]. This variability may also depend on the experimental conditions, such as the pH, oxygen availability, medium composition, or temperature [39]. It is important to consider that OD measurements, while useful, do not differentiate between viable and dead cells, potentially leading to an overestimation of biomass, particularly in the stationary phase. The exponential phase, characterized by high metabolic activity, could correspond to the peak virulence factor production, whereas the stationary phase may depict the presence of resilience mechanisms, such as those described in biofilm formation or dormancy. These conditions, therefore, may influence not only the bacterial activity and their virulence factor production, but also the inflammatory response elicited by the macrophages.

Macrophages play a crucial role in the innate immune response, being one of the first immune cells to be recruited at the site of infection [24,25]. Because of their limited life span and high variability demonstrated by human macrophages ex vivo, the human leukemia-derived monocytic cell line THP-1 is the most widely used cell line to investigate in vitro the function of primary human macrophages. The phorbol 12-myristate 13-acetate (PMA), an activator of protein kinase C, has been commonly used to induce THP-1 towards a macrophage-like phenotype, although there is not a standardized protocol and different studies have reported a variety of conditions ranging from different concentrations of PMA and diverse exposure times or recovery periods [40]. For this reason, we optimized the experimental conditions for the macrophage polarization using two strategies, one using direct stimulation with increasing concentrations of *P. gingivalis* LPS, activating the classical pathway through Toll-like Receptors (TLR) 2 and TLR-4 [27,41]; while the other used recombinant human IFN-γ (20 ng/mL), which induces M1 macrophage subpopulations via the STAT6 pathway. Lastly, a combination of LPS and IFN-γ was used to investigate their combined effects on macrophage activation [42,43]. It was demonstrated that the LPS from *P. gingivalis* W83 induced a strong inflammatory response, characterized by the increase in production of pro-inflammatory cytokines such as TNF-α, IL-1β, and IL-6, and this release was enhanced when IFN-γ was added. For this reason, we considered using unprimed macrophages to implement the infection model. The observed upregulation of pro-inflammatory cytokines underscores their pivotal role in driving in vivo chronic inflammation and tissue destruction in periodontitis. These cytokines amplify the inflammatory cascade by recruiting immune cells, such as neutrophils and monocytes, to periodontal tissues, while also stimulating matrix metalloproteinases (MMPs), which degrade the extracellular matrix and contribute to alveolar bone resorption [1,2]. Interleukin-6, beyond its role in acute inflammation, facilitates the transition to chronic inflammation by promoting Th17 differentiation and sustained cytokine production [44,45]. Together, these cytokines perpetuate a destructive inflammatory microenvironment conducive to bacterial persistence and tissue degradation. The inability of the immune system to effectively eliminate *P. gingivalis* due to its immune-evasive strategies, such as complement degradation and disruption of phagocytosis, further exacerbates inflammation. This creates a feedback loop where cytokine overproduction and unresolved inflammation promote progressive tissue destruction [6,46]. 

M1 macrophages aim to eliminate pathogens; however, in the context of periodontitis, their prolonged activation inadvertently damages host tissues, including periodontal ligaments and alveolar bone [47,48]. *Porphyromonas gingivalis* subverts macrophage function by disrupting phagosome maturation, evading reactive oxygen species (ROS)-mediated killing and impairing the natural resolution of inflammation. Specialized pro-resolving lipid mediators derived from omega-3 fatty acids play a crucial role in counteracting such disruptions by actively promoting the resolution of inflammation and restoring tissue homeostasis [49]. However, in the presence of *P. gingivalis*, this balance is disrupted, allowing the pathogen to persist and skew macrophage polarization towards an M1-dominant state, fueling chronic inflammation [34].

The amount of *P. gingivalis* detected in this experimental model provided critical information on the effectiveness of immune defense mechanisms and the pathogen’s evasion strategies. The present study showed that low levels of *P. gingivalis* (only 3.40 × 10^3^ CFU/mL) were either internalized or adherent to macrophages after 1 h exposure, which coincides with the findings of studies by Werheim et al., who reported that the *P. gingivalis* W83 strain exhibited an escape rate exceeding 60% of total intracellular CFUs, thus demonstrating the highly effective mechanisms of this pathogen to avoid macrophage-mediated killing [15]. Other studies have also shown that *P. gingivalis* predominantly remains extracellular, thus avoiding its internalization [16,50]. In this study, the detection of low levels of adherent or internalized bacteria after one hour of exposure can be interpreted in several ways: *P. gingivalis*, particularly the W83 strain, has developed multiple mechanisms to avoid phagocytosis or elimination by macrophages. Its ability to inhibit phagosome maturation or escape from it is well-documented [28,34]. Therefore, the low levels detected could reflect that the bacterium effectively avoids internalization and uses strategies to remain predominantly in the extracellular environment. Alternatively, experimental conditions might influence bacterial survival. For instance, using an aerobic environment, when *P. gingivalis* is an obligate anaerobe, could reduce its proliferation capacity. This highlights the need to validate experimental models to ensure they replicate relevant physiological conditions.

The cytokine levels and the amounts of *P. gingivalis* detected were intrinsically related: low bacterial levels accompanied by high cytokine concentrations could indicate a hyperreactive immune response that, while failing to eliminate the pathogen, causes significant collateral damage to host tissues.

This infection model, however, may have relevant limitations. One could be the selection of *P. gingivalis* W83, which was based on the well-demonstrated virulence of this strain, which would likely elicit a strong macrophage response—but it is not, however, the predominant strain found in most patients. Therefore, this model will need to be tested with other bacterial strains, such as *P. gingivalis* ATCC 33277. Additionally, in vitro models cannot fully replicate the complex physiological conditions of a living organism. The use of macrophages derived from cell lines, such as THP-1, may not accurately reflect the behavior of primary human macrophages, and the polarization state (M1/M2) might not adequately represent the in vivo conditions. While monocytic cell lines, such as U937, HL-60, or THP-1 cells, are widely used in biomedical research as precursors of human macrophages for their consistent genetic background and ease of handling, they do not fully replicate the phenotypic and functional heterogeneity of primary human macrophages. Moreover, the malignant phenotype of cell lines has been shown to be associated with different responsiveness in terms of gene expression changes compared to primary macrophage [50,51]. Macrophages derived from primary human peripheral blood monocytes represent an alternative that offers greater physiological relevance. However, a limited lifespan and the inter-individual donor-dependent variability are the main disadvantages for implementing studies to investigate the innate immune response in vitro [52]. Considering these limitations, further research on the validation of the THP-1 cell line as a source of macrophages might be considered using more complex and physiologically relevant systems to enhance translational potential [53,54].

To elucidate the molecular mechanisms driving *P. gingivalis*-induced inflammation and immune evasion, the integration of multi-omics approaches could be considered. Combining these datasets within a systems biology framework could help identify key molecular pathways, regulatory networks, and metabolic weaknesses as potential therapeutic targets.

## 4. Materials and Methods

### 4.1. Microbial Growth Curve Optimization

Growth curve: The standard reference strain *P. gingivalis* W83 ATCC BAA-308™ (Manassas, VA, USA) was grown on blood agar plates (Blood Agar Oxoid No 2; Oxoid, Basingstoke, UK) supplemented with 5% (*v*/*v*) sterile horse blood (Oxoid, Basingstoke, UK), 5.0 mg/L haemin (Sigma, St. Louis, MO, USA), and 1.0 mg/L menadione (Merck, Darmstadt, Germany) at 37 °C for 48–72 h under anaerobic conditions (10% H_2_, 10% CO_2_, and 80% N_2_). A single colony of *P. gingivalis* W83 was isolated and transferred to a 25 mL liquid medium containing brain-heart infusion (BHI) (Becton, Dickinson and Company, Franklin Lakes, NJ, USA) supplemented with 5.0 mg/L hemin and 1.0 mg/L menadione and incubated overnight (16–18 h). Optical density (OD) was measured at 550 nm and adjusted at 0.2 OD by adding fresh medium. During 12 h, OD measurements were recorded every 2 h. From each sampling point, 1:10 serial dilutions were prepared and 100 µL of each selected dilution was plated and incubated for 7 days. Colony-forming units American Type Culture Collection (ATCC), Manassas, Virginia, USA per milliliter (CFU/mL) were calculated for each sampling point. Three independent replicates of these experiments were performed to establish the growth curve and identify the exponential phase.

Quantitative PCR (qPCR) standard curve: Ten-fold serial dilutions of purified genomic DNA were prepared from a *P. gingivalis* W83 sample with an initial concentration of 5.32 × 10^9^ CFU/mL. These dilutions were used to construct a qPCR standard curve to ensure assay linearity and accurate quantification. DNA extraction was performed using the MolYsis™ Complete5 kit (Molzym, Bremen, Germany), following the manufacturer’s protocol, which includes steps for selective lysis of host cells, bacterial cell lysis, and removal of PCR inhibitors. Primers and probes were based in previously published information [55]: Primer F: 5′-GCGCTCAACGTTCAGCC-3′; Primer R: 5′-CACGAATTCCGCCTGC-3′; Probe: 5′-6 FAM-CACTGAACTCAAGCCCGGCAGTTTCAA-TAMRA-3′ (Invitrogen and Applied Biosystems, Waltham, MA, USA). Amplification was conducted in a total reaction volume of 10 µL, containing 5 µL of 2× Master Mix (LC 480 Probes Master, Roche), primers and probes at 300 nM, and 2.5 µL of extracted DNA. Negative control consisted of 2.5 µL sterile water (non-template control (NTC)) (Water PCR grade, Roche, Basel, Switzerland). The amplification program included an initial cycle at 95 °C for 10 min, followed by 45 cycles at 95 °C for 15 s and 60 °C for 1 min, run on a LightCycler 480 II (Roche). qPCR plates used were LightCycler 480 Multiwell-384 (Roche). Each DNA sample was analyzed in duplicate. The resulting qPCR standard curve evaluated the relationship between the Cq value and the logarithm of DNA concentration, ensuring reproducibility [56,57,58]. The efficiency of the qPCR was calculated from the slope of the standard curve using the formula 10^(−1/slope)^, with optimal performance expected in the range of 90–100% efficiency (−3.6 ≤ slope ≤ −3.3). This step ensured that the amplification reactions were highly efficient, supporting the robustness and reproducibility of the assay for downstream analyses.

### 4.2. Macrophage Cell Culture

Macrophages were derived from the human monocyte cell line THP-1, originally isolated from a patient with acute monocytic leukemia, obtained from ATCC TIB-202 (Manassas, VA, USA). The vial contents were transferred to a centrifuge tube containing 9.0 mL of complete growth medium and centrifuged at 125 g for 5 min. The base medium, RPMI-1640 (ATCC formulation, Manassas, VA, USA), was supplemented with 10% fetal bovine serum (FBS) and 1% antibiotics (100 U/mL penicillin and 100 μg/mL streptomycin, GIBCO) to create the complete growth medium. Before adding the cells, the culture vessel with complete medium was placed in the incubator for at least 15 min to stabilize the pH (7.0 to 7.6). The cell pellet was then resuspended in the medium and cultured in upright Corning^®^ T-25 flasks. Cultures were maintained by refreshing or replacing the medium every 3 days to keep cell concentrations below 1 × 10^6^ cells/mL. A cell stock was generated from subcultures and stored in nitrogen using FBS with 10% DMSO as a cryopreservative. A freeze–thaw test was performed to ensure cell viability while other cells remained in culture. For experiments, the same initial culture protocol was used, with medium refreshed every 3 days. Cell concentration and viability was checked using the Trypan Blue exclusion assay using an haematocytometer.

To induce macrophage activation, a stock solution of phorbol 12-myristate 13-acetate (PMA) (Sigma-Aldrich, St. Louis, MO, USA) was prepared in DMSO at 1 mg/mL. THP-1 cells were exposed to PMA concentrations ranging from 25 to 100 ng/mL in complete medium for 24–72 h. Differentiation was confirmed by observing cell adherence to the plate surface using a Nikon ECLIPSE TS 100 inverted microscope with 10× and 20× objectives.

To induce M1 macrophage polarization, cells were stimulated with *P. gingivalis* lipopolysaccharide (LPS) (SMB00610 Sigma-Aldrich, St. Louis, MO, USA) at different concentrations alone or combined with 20 ng/mL recombinant human IFN-γ (Gibco). THP-1 cells were seeded in 48-well plates at a density of 4 × 10^5^ cells/mL and treated with 25 ng/mL PMA for 48 h to induce differentiation. Subsequently, LPS at three concentrations (1,10, and 100 ng/mL) was added either alone or combined with IFN-γ (20 ng/mL). After 24 h incubation, the culture supernatants were collected and analyzed for selected cytokines measurement using Luminex^®^ technology, either immediately or after storage at −80 °C.

#### Measurement of Cytokines

The bead-based fluorescence assay was the MILLIPLEX^®^ Human Cytokine/Chemokine/Growth Factor Panel, a magnetic bead panel (Product #HCYTA-60k, Merck KGaA, Darmstadt, German) for simultaneous evaluation of four cytokines (TNF-α, interleukin [IL]-1β, IL-6 and IL-4). This assay was run on the Luminex^®^ 200^TM^ detection instrument operated with xPONENT Software V4.3 (both from Luminex^®^ Corp., Austin, TX, USA). All assay steps were performed in accordance with manufacturer instructions. Samples, kit standards, and controls were manually pipetted into assay plates, while reagents dispensation was performed using a multichannel electronic pipette. All samples were assayed in duplicate. Washing steps were performed using an automated programmable washer-dispenser (BioTek 50 TS Microplate Washer). Analyte concentrations were determined by comparing sample readings to standard curves generated using a 4-parameter logistical curve fit algorithm (Belysa v.1 software from Millipore/Sigma). Two independent experiments were performed: the first used frozen samples and the second used fresh samples. Due to methodological differences that could influence the magnitude of the outcomes, the results were analyzed separately, and no means were calculated. Analyte concentrations, expressed as pg/mL, are presented as means with standard deviations of two measurements.

### 4.3. Infection Protocol of Activated Macrophages with P. gingivalis W83

THP-1 cells were counted and seeded at a density of 2 × 10^6^ cells per T25 flask (5 × 10^5^ cells/mL). To induce differentiation, THP-1 cells were stimulated 72 h prior to the experiment. PMA was added at 25 ng/mL to the medium for 48 h, then it was removed, and complete RPMI medium without PMA was replaced, allowing the cells to differentiate into M0 macrophages. *Porphyromonas gingivalis* W83 was harvested during the exponential growth phase. The bacteria were diluted 1:10 in RPMI medium without antibiotics and FBS and added to M0 macrophages in triplicate.

After 1 h of incubation at 37 °C and 5% CO_2_, the supernatants were removed from both the negative controls (mock infected) and the infected macrophages and centrifuged at 13,000 rpm for 3 min. The resulting pellets were stored at −20 °C for later DNA extraction and qPCR analysis.

Next, both mock and infected macrophages were dissociated and detached from the flasks through incubation with TrypLE (Gibco) for 10–15 min at 37 °C followed by scraping. The cellular suspension was centrifuged at 1300 rpm for 5 min to prepare the resulting cell pellet for lysis according to the instructions of the MolYsis Complete5 kit (Molzym, Bremen, Germany) for bacterial DNA extraction. Quantitative PCR assays were performed as previously described. The chart in Figure 11 shows the distribution of *P. gingivalis* bacteria after 1 h of incubation, with error bars representing the standard deviation of three biological replicates (*n* = 3).

### 4.4. Statistycal Analysis

Quantitative data are expressed as colony-forming units per milliliter (CFU/mL), optical density (OD) at 550 nm. Results are reported as means with standard deviations (SDs), and data normality was assessed using the Shapiro–Wilk goodness-of-fit test.

A linear regression was performed to analyze the correlation between OD and CFU/mL of the growth curve. The scatter plot includes the regression line derived from the data, along with the coefficient of determination (R^2^), which quantifies the proportion of variance explained by the model. The *p*-value was calculated to assess the statistical significance of the relationship.

An exponential fit was applied to the growth curve to distinguish and characterize the distinct bacterial growth phases. The growth data were segmented into four key phases: lag phase, exponential phase, stationary phase, and death phase. In the exponential phase, the data points were fit to an exponential growth model (*OD*(_*t*_) = *OD*(_*t*0_) · *e*^*k*·*t*^) [59] to quantify the increase in bacterial population. The specific growth rate (*k*) was calculated to determine the dynamics of bacterial replication.

Two independent experiments were conducted to analyze cytokine production by macrophages. In the first experiment, samples were frozen at −80 °C before analysis, while in the second experiment, fresh samples were used without prior freezing. Due to methodological differences, the results were analyzed separately, and no means were calculated. Analyte concentrations (pg/mL) are presented as means of two technical replicates. Additionally, the fold change in relative cytokine expression is presented, with the data normalized to the baseline group (NS48 h_24 h rest).

GraphPad Prism version 8.0.1 software was used for all data analysis.

## 5. Conclusions

This study demonstrates that *P. gingivalis* W83 is able to induce the inflammatory M1 phenotype of THP-1-differentiated macrophages with an increased production of TNF-α, IL-1β, and IL-6. After one hour of incubation of *P. gingivalis* with unprimed macrophages, similar levels of bacterial cells were detected in the medium when compared to the initial inoculum, demonstrating the ability of this strain to probably escape the phagocytosis by macrophages contributing to chronic inflammation in periodontitis. These findings provide valuable insights into the immune response, suggesting that future research integrating genomic and multi-omics approaches could identify new therapeutic targets for periodontitis.

## Figures and Tables

**Table 1:** Growth curve counts. Data are presented as means (standard deviations).

T (hours)	OD (550 nm) (SD)	CFU/mL (SD)
0 (0 h)	0.233 (0.038)	3.70 × 10^8^ (6.52 × 10^7^)
1 (2 h)	0.248 (0.022)	4.75 × 10^8^ (1.43 × 10^8^)
2 (4 h)	0.327 (0.017)	6.43 × 10^8^ (9.55 × 10^7^)
3 (6 h)	0.487 (0.043)	9.43 × 10^8^ (3.47 × 10^8^)
4 (8 h)	0.674 (0.035)	9.80 × 10^8^ (2.62 × 10^8^)
5 (10 h)	0.870 (0.039)	1.29 × 10^9^ (4.93 × 10^8^)
6 (12 h)	1.042 (0.043)	1.21 × 10^9^ (6.02 × 10^8^)

T: hours, OD: optical density, CFU/mL: colony-forming units/mL, SD: standard deviation.

**Table 2:** Fold change in the relative amount of pro-inflammatory cytokines IL-1β, TNF-α, and IL-6. The data are normalized relative to the baseline group (NS48 h_24 h rest). Values marked with * represent data from a second independent experiment.

	IL-1 β (pg/mL)	TNF-α (pg/mL)	IL-6 (pg/mL)
NS 48 h_24 h rest (Baseline)	1.00	1.00	1.00
IFN-γ	1.25	0.86	1.06
*	1.66	4.44	5.79
LPS 1	1.07	1.06	0.94
*	1.33	1.46	7.88
LPS 10	3.57	6.83	3.29
*	2.27	3.47	3.90
LPS 100	10.31	23.91	13.34
*	3.84	10.81	9.53
LPS 1_IFN- γ	2.09	2.81	2.79
*	1.56	2.43	17.54
LPS 10_IFN- γ	5.84	12.86	11.42
*	2.99	7.80	1.89
LPS 100_IFN- γ	13.26	41.83	49.93
*	4.38	17.55	8.26

L-1β: Interleukin-1 beta; TNF-α: Tumor Necrosis Factor-alpha; IL-6: Interleukin-6; NS48 h_24 hrest: Non-stimulated, baseline; IFN-γ (Interferon-gamma): IFN-γ 20 ng/mL; LPS (Lipopolysaccharide) 1: LPS 1 ng/mL; LPS 10: LPS 10 ng/mL; LPS 100: LPS 100 ng/mL; LPS 1_IFN-γ: LPS 1 ng/mL + IFN-γ 20 ng/mL; LPS 10_ IFN-γ: LPS 10 ng/mL + IFN-γ 20 ng/mL; LPS 100_ IFN-γ: LPS 100 ng/mL + IFN-γ ng/mL.

## Data Availability

Data are contained within the article.
